# Impact of N, P, K, and Humic Acid Supplementation on the Chemical Profile of Medical Cannabis (*Cannabis sativa* L)

**DOI:** 10.3389/fpls.2019.00736

**Published:** 2019-06-17

**Authors:** Nirit Bernstein, Jonathan Gorelick, Roei Zerahia, Sraya Koch

**Affiliations:** ^1^ Institute of Soil Water and Environmental Sciences, Volcani Center, Rishon LeZion, Israel; ^2^ Eastern Regional R&D Center, Kiryat Arba, Israel; ^3^ Pernick Faculty of Engineering, Shenkar College of Engineering and Design, Ramat Gan, Israel; ^4^ The Robert H. Smith Faculty of Agriculture, The Hebrew University of Jerusalem, Rehovot, Israel

**Keywords:** cannabis, cannabinoid, THC, CBD, fertilizer, humic acid, nutrition, nitrogen

## Abstract

Mineral nutrition is a major factor affecting plant growth and function. Increasing evidence supports the involvement of macro and micronutrients in secondary metabolism. The use of the appropriate nutritional measures including organic fertilizers, supplements, and biostimulants is therefore a vital aspect of medicinal plant production including medical cannabis. Due to legal restriction on cannabis research, very little information is available concerning the effects of nutritional supplements on physiological and chemical properties of medical cannabis, and their potential role in standardization of the active compounds in the plant material supplied to patients. This study therefore evaluated the potential of nutritional supplementations, including humic acids (HAs) and inorganic N, P, and K to affect the cannabinoid profile throughout the plant. The plants were exposed to three enhanced nutrition treatments, compared to a commercial control treatment. The nutrition treatments were supplemented with HA, enhanced P fertilization, or enhanced NPK. The results demonstrate sensitivity of cannabinoids metabolism to mineral nutrition. The nutritional supplements affected cannabinoid content in the plants differently. These effects were location and organ specific, and varied between cannabinoids. While the P enhancement treatment did not affect THC, CBD, CBN, and CBG concentrations in the flowers from the top of the plants, a 16% reduction of THC concentration was observed in the inflorescence leaves. Enhanced NPK and HA treatments also produced organ-specific and spatially specific responses in the plant. NPK supplementation increased CBG levels in flowers by 71%, and lowered CBN levels in both flowers and inflorescence leaves by 38 and 36%, respectively. HA was found to reduce the natural spatial variability of all of the cannabinoids studied. However, the increased uniformity came at the expense of the higher levels of cannabinoids at the top of the plants, THC and CBD were reduced by 37 and 39%, respectively. Changes in mineral composition were observed in specific areas of the plants. The results demonstrate that nutritional supplements influence cannabinoid content in cannabis in an organ- and spatial-dependent manner. Most importantly, the results confirm the potential of environmental factors to regulate concentrations of individual cannabinoids in medical cannabis. The identified effects of nutrient supplementation can be further developed for chemical control and standardization in cannabis.

## Introduction

*Cannabis sativa* has been used for medical purposes in traditional medicine since antiquity and is currently being evaluated as a promising treatment for a wide range of medical indications (Grotenhermen and Müller-Vahl, 2012; Alexander, 2016). The pharmaceutical activity of cannabis is attributed to hundreds of secondary metabolites, including cannabinoids, terpenes, and flavonoids, which are produced mainly in female flowers (ElSohly and Gul, 2014; Hanuš et al., 2016; Gorelick and Bernstein, 2017; Shtein and Bernstein, 2018, submitted). For utilization in modern medicine, the composition and concentrations of these compounds in the plant material supplied to patients need to be standardized (Gorelick and Bernstein, 2017). Understanding the regulation of the biosynthesis and accumulation of the secondary metabolites in the various plant organs is thereby required.

The content and composition of secondary metabolites in plants is affected by both genetics and environmental factors (Gorelick and Bernstein, 2017). While genetics determine the potential for production, environmental conditions induce variations in quantity, quality, and distribution of the active compounds in the plant. Secondary metabolite profile is thereby a result of the interaction of environmental and physiological processes. Currently, due to legal restrictions of cannabis research, we lack basic information regarding plant biosynthetic regulation. Moreover, there is very little knowledge and understanding of the interrelations between chemistry and environmental effects in cannabis. Soil fertility and mineral nutrition are major environmental factors affecting plant development, function, and metabolism. Nitrogen (N), phosphorus (P), and potassium (K) are the three most abundantly acquired mineral elements by plants, and they play vital roles in many aspects of plant metabolism. There is some evidence supporting the influence of mineral nutrition, and especially the major macronutrients N, P, and K on secondary metabolites. Macronutrients were reported to affect the terpene profile in aromatic plants (Piccaglia et al., 1989; Rioba et al., 2015), and there are conflicting reports concerning the effects of P and N supplementation on numerous secondary metabolites including flavonoids, glucosinolates and phenylpropanoids, biosynthesized from the amino acids phenylalanine and tyrosine (Dixon and Paiva, 1995; Jeliazkov and Margina, 1996; Arabaci and Bayram, 2004; Barreyro et al., 2005; Nell et al., 2009; Pant et al., 2015; Rioba et al., 2015). Variations in micronutrients and soil salinity can also affect the secondary metabolite profile (Singh and Misra, 2000; Bernstein et al., 2010). While emphasis is usually placed on the availability of sufficient quantities of the major plant nutrients, the potential biostimulant role of nutritional supplementation must be considered as well.

Physical and chemical conditions in the soil often restrict nutrient availability for plant uptake. Plant biostimulants, which have the capacity to indirectly affect nutrient availability and uptake and modify physiological processes in plants, are therefore becoming increasingly popular (du Jardin, 2015). Biostimulants can be produced from a number of organic or microbial sources and have been shown to improve soil structure, root development, and nutrient uptake in a number of important agricultural crops. While they are utilized extensively in agriculture to increase yield, disease, and drought resistance, their usage in the production of medicinal plants is more complex. There is a widespread belief that plants grown in organic settings are richer in secondary metabolites than traditionally grown plants (Adam, 2001). However, there is little evidence to support this claim.

A popular plant biostimulant is humic acid (HA), an organic soil amendment attributed with growth-stimulating activity (Peña-Méndez et al., 2005). HA is derived from humic substances, known as humus, a microbial metabolized organic matter which comprises over 60% of the organic soil matter in the world (Muscolo et al., 2013). While HA is known as a fertilizer or nutritional supplement, it is on a more basic level, a soil amendment, improving the physical and chemical properties of the soil, affecting soil pH and increasing moisture and nutrient availability (Gümüş and Şeker, 2015). As a biostimulant, HA also affects plant growth and development directly *via* nutritional, hormonal, or elicitory pathways (Zandonadi et al., 2007; Billard et al., 2014; Canellas and Olivares, 2014; Conselvan et al., 2017). Therefore, it is not surprising that in addition to its primary role in nutrient uptake, HA is also involved in secondary metabolite biosynthesis. This influence was clearly demonstrated in roots, where humic substances enhanced the exudation of various organic acids (Canellas et al., 2008). But this effect is not only relegated to roots. HA was shown to enhance phenlypropanoid biosynthesis in maize (Schiavon et al., 2010). These findings have led many to believe that HA supplementation can enhance the biosynthesis of therapeutic secondary metabolites in medicinal plants. This is especially the case with cannabis, were HA is claimed to increase production and a number of HA-based products are marketed for cannabis cultivation. While there is some evidence supporting the beneficial aspects of humic acid in cannabis cultivation (Ievinsh et al., 2017), its effects on cannabinoid content have yet to be studied.

While it is clear that mineral nutrition and nutritional supplements, which are known to influence all major physiological process, should also affect secondary metabolism, there is very little work characterizing this connection. In the case of cannabinoid production in medical cannabis, almost no work has been performed documenting the effects of mineral nutrition and nutritional supplements on cannabinoid content.

In this study, we therefore focused on the chemical and physiological responses of medical cannabis to N, P, K, and HA supplements. The present study aimed to check potential effects of the supplemented nutrients under what is currently considered an optimal range of these nutrients supply. We aimed to see if alteration of the supply, without harming the plants by imposing deficiencies or toxicities, affects cannabinoid regulation. The present study was thus undertaken to evaluate the following hypotheses: (1) nutritional supplementations of humic acids and inorganic N, P, K under conditions of optimal fertilization elicit changes in the cannabinoid profile of medical cannabis; (2) the elicited changes are organ dependent (i.e., flowers, fan leaves, inflorescence leaves) and spatially dependent in the plant; (3) the elicited changes are associated with changes to the physiological state of the tissue, and the tissue ionome. To test these hypotheses, we studied effects of the nutritional supplementations on: (1) cannabinoid composition and concentration, (2) ionome, and (3) physiological characteristics of cannabis plant organs.

## Materials and Methods

### Plant Material and Growing Conditions

The medical cannabis (*Cannabis sativa*) cultivar “NB100” (CANNDOC LTD, Israel), which is one of the cultivars approved for medical use in Israel, was used as a model system in this study. It is a high THC variety, with indica characteristics. Plants were propagated from cuttings of a single mother plant in coconut fiber mixture. Rooted cuttings were planted in 4.5-L black plastic pots in a potting mixture, and cultivated under 18/6-h light/dark photoperiod in a commercial medical cannabis farm in a greenhouse equipped with an evaporative cooling system (CANNDOC LTD, Israel). After 3 weeks, when the plants reached 25 cm in height, they were transferred to a 12/12-h short day photoperiod for an additional 8.5 weeks to induce flowering after which all plant material was collected for analysis. Cultivation was conducted under sunlight. When needed, artificial illumination by 20-W PL fluorescent lamps was used to extend the photoperiod. Maximum and minimum temperatures in the greenhouse were 26 and 18°C day/night. Minimum day and maximum night relative humidities were 60 and 90%, respectively. Irrigation was supplied *via* 1.2 L h^−1^ discharge-regulated drippers (Plastro Gvat, Israel), 1 dripper per pot. Each irrigation pulse was 500–800 ml/pot, one pulse per day, set to allow 25% of drainage. Plant density was 2 plants per m^2^.

### Treatments

The plants were exposed to three enhanced nutrition treatments, compared to a commercial [control] treatment. The enhanced nutrition treatments received the control treatment with the addition of either humic acids [+HA]; enhanced P fertilization [+P]; or enhanced NPK treatment [+NPK]. The fertilizers were supplied by fertigation, i.e., dissolved in the irrigation solution at each irrigation event at concentrations of 65 ppm N (with 1:2 ratio of NH_4_^+^ / NO_3_^–^), 40 ppm P_2_O_5_ (17 ppm P), and 108 ppm K_2_O (90 ppm K). Micronutrients were supplied chelated with EDTA at concentrations of 0.4 ppm Fe, 0.2 ppm Mn, and 0.06 ppm Zn. Fertilization was conducted from pre-mixed (final) solutions. For the [+HA] treatment, humic acids were added daily, 2 h after the last fertilization each day, as a liquid humic acid solution, 200 ml/pot of a 1:10 (W/W) dilution of a commercial product containing 12% humic acid (Uptake 12, Lidorr chemicals LTD, Ramat Hasharon, Israel). The remaining treatments received the same volume of irrigation without the addition of HA. No leachates were produced following this addition. The [+P] treatment was supplemented with 10 g 20% superphosphate (Ca(H_2_PO_4_)_2_)/pot (ICL, Haifa, Israel) at the transition to the flowering photoperiod and every 3 weeks thereafter. The fertilization solution of the [+NPK] treatment was supplemented with 15% higher concentrations of N, P, and K than the control treatment, added as KNO_3_, NH_4_NO_3_, and H_3_PO_4_ to the final concentrations of 75, 20, and 104 ppm N, P, and K, respectively. Fertigation was managed in an open cycle.

### Sampling Plant Material

The plants were sampled for cannabinoid quantification, inorganic mineral analysis, and physiological parameters analyses after reaching the maturity stage acceptable for the commercial harvest of medical cannabis, i.e., 50% of the trichomes on the inflorescences were of amber color, 8.5 weeks after they were transferred to the flowering-induced photoperiod.

### Cannabinoid Quantification

Cannabinoid concentrations were analyzed in flowers and inflorescence leaves from three different heights of the plants, and in fan leaves. The tissue analyzed was the apical 2 cm of the largest inflorescence from the top of the plant [top], the apical inflorescence of a side branch terminating at mid-height of the plant [center], and an inflorescence from the bottom of a side branch [bottom]. The sampled inflorescences were then separated into flowers and inflorescence leaves and were dried at 16–18°C and 55% relative humidity for 3 weeks before further analyses. Fan leaves analyzed were from the top part of the main branch.

A total of 20 mg of ground dried plant material was extracted with 2-ml absolute ethanol, cellulose filtered, and diluted with an internal standard (tetracosane, 50 μg/ml) to a final concentration of 1 mg/ml. Samples (1 μl) were injected into a GC-MS (Hewlett Packard G 1800B GCD system) running GCD Plus Chemstation (Palo Alto, USA). A SPB-5 column (30 m × 0.25 mm × 0.25 μm film thickness) was used under the following initial conditions: inlet temperature of 250°C; detector temperature of 280°C; and a helium flow rate of 1 ml/min. The initial temperature (100°C) was held for 2 min and then raised at a rate of 10°C/min until a final temperature of 280°C was reached. Standard curves for each of the cannabinoids studied were generated using standards of each cannabinoid at increasing concentrations ranging from 1 to 1,000 μg/ml together with 50.0 μg/ml tetracosane as an internal standard.

### Inorganic Mineral Analysis

For the analyses of inorganic mineral content in the plant, the plants were destructively harvested and each plant was separated into: flowers from large inflorescences (longer than 5 cm – found at the top of the main branches), flowers from the remaining smaller inflorescences, fan leaves, inflorescence leaves, and stems. Three different procedures were applied for extraction of the various inorganic mineral elements from the plant tissue (Sacks and Bernstein, 2011). For the analysis of N, P, and K, the dry tissue was digested with H_2_SO_4_ (98%) and H_2_O_2_ (70–72%). K was analyzed by a flame photometer (410 Flame Photometer Range, Sherwood Scientific Limited, The Paddocks, UK), and P and N by an autoanalyzer (Lachat Instruments, Milwaukee, WI, USA). For the analyses of Cl, dried plant samples were extracted with a dilute acid solution containing 0.1 N HNO_3_. Cl was measured by potentiometric titration (PCLM3 Jenway, Bibby Scientific Ltd., T/As Jenway, Dunmow, UK) (Bernstein et al., 2017). For the analysis of Ca, Mg, Fe, Zn, Mn, and Cu, the dry tissue was digested with HNO_3_ (65%) and HClO_4_ (70%), and the elements were analyzed with an atomic absorption spectrophotometer, AAnalyst 400 AA Spectrometer (PerkinElmer, Massachusetts, USA). All analyses were conducted with 5-point calibration curves.

### Determination of Membrane Leakage

Ion leakage from leaf tissue is considered an indicator of membrane injury under stress. Leakage often increases under exposure to biotic and abiotic stresses including mineral toxicities and deficiencies due to increased lipid peroxidation by increased free radical production. In the present study, membrane leakage measurements were aimed to evaluate if the tissue suffered stress due to the higher concentrations of solutes applied to the root zone. It was measured as previously described with minor modifications (Shoresh et al., 2011). The youngest mature leaf on the plant was carefully removed and washed twice in sterilized distilled water. The leaf petiole, mid-rib, and leaflet margins were removed with the aid of a scalpel. The remaining leaf tissue segments were transferred to a 50-ml tube with 30 ml of double-distilled water and shaken for 24 h, or sampled for osmotic potential determination. The electric conductivity (EC) was measured using a conductivity meter Cyberscan CON 1500 (Eutech Instruments Europe B.V. Nijkerk, Netherlands). Then, the samples were autoclaved for 30 min to destroy cells and cause 100% leakage. The autoclaved samples were allowed to cool down for 45 min and were re-shaken for an additional 1 h. The EC was re-measured. Ion leakage from the plant tissue was calculated as percent (%) of EC value before autoclaving to its value post autoclaving. Results from six replicated leaves from six replicated plants were averaged.

### Determination of Osmotic Potential

Osmotic potential of the tissue sap is a measure of total solute concentration. It often increases under water, salinity, or toxic stress due to elevated uptake and accumulation, tissue drying, or osmotic adjustment. The measurements in the present study were aimed to evaluate if the increased concentration of solutes in the nutrient supplementation treatments increased accumulation or imposed osmotic stress. For osmotic potential measurements, the sampled tissue was frozen in 1.5-ml micro test tubes in liquid nitrogen and stored at −20°C for further analyses. The frozen tissue was crushed inside the tubes with a glass rod, the bottom of the tubes was pin-pricked and the tubes, set inside another 1.5-ml tube, were centrifuged for 4 min in a refrigerated centrifuge (Sigma Laboratory Centrifuges, Germany) at 4°C at 7,000 rpm. Fifty microliters of fluid collected in the lower tube were used for measurement of osmotic potential using a cryoscopic microosmometer Osmomat 3,000 (Gonotec, Berlin, Germany) by measuring the freezing point of 50 μl of sap. Results are presented in mOsm kg^−1^ H_2_O^−1^. Six replicated leaves from six replicated plants were analyzed.

### Determination of Chlorophyll and Carotenoid Content

The youngest mature fan leaf on the plant was separated from the rest of the shoot and rapidly washed in distilled water. A 20-mm segment of tissue located half way along the length of the central leaflet was used for chlorophyll and carotenoid analysis. Five discs, 0.6 cm in diameter, were cut from this leaf section avoiding the mid-rib, placed in 0.8 ml 80% (v/v) ethanol, and heated to 92°C for 30 min. The soluble boiled extract was collected in 2-ml micro test tubes. The remaining tissue was extracted again in 0.5 ml 80% (v/v) ethanol for 15 min at room temperature and the combined extract was mixed by vortex. Next, 0.4 ml of extract was mixed with 5 ml 80% (v/v) acetone, and absorbance at 663, 646, and 470 nm was measured using a Genesys 10 UV Scanning spectrophotometer (Thermo scientific). Calculation of chlorophyll a and b and carotenoids was done according to Lichtenthaler and Wellburn (1983). Reported results are averages of six replicated leaves from six replicated plants.

### Plant Architecture and Development

After 8.5 weeks of the transition of the plants to the flowering-induced photoperiod, in parallel to the sampling for chemical analyses, the plants were harvested destructively and sampled for morphological analyses. Plant height, stem diameter as well as the number of side branches and internodes on the main stem were measured. Plant height was measured from the base of the plant to the top branch and stem diameter was measured with a digital caliper 10 cm from the plant base. The measurements were conducted on six replicated plants per treatment.

At the time of the destructive harvest, the shoot was separated into fan leaves, inflorescence leaves, stems, and flowers, and the distribution of plant biomass between these vegetative and reproductive organs was evaluated. Fresh biomass was measured immediately following sectioning and dry weights were measured following desiccation at 64°C. Presented results are averages ± SE for six replicated plants.

### Experimental Design and Statistics

The experiment was set in a “completely randomized design,” with four treatments and six replicated plants per treatment. Each plant constituted a replicate. The data were subjected to ANOVA followed by Tukey’s HSD test. The analysis was performed with the Jump software (Jump package, version 9, SAS 2015, Cary, NC, USA).

## Results

**Figure 1:**
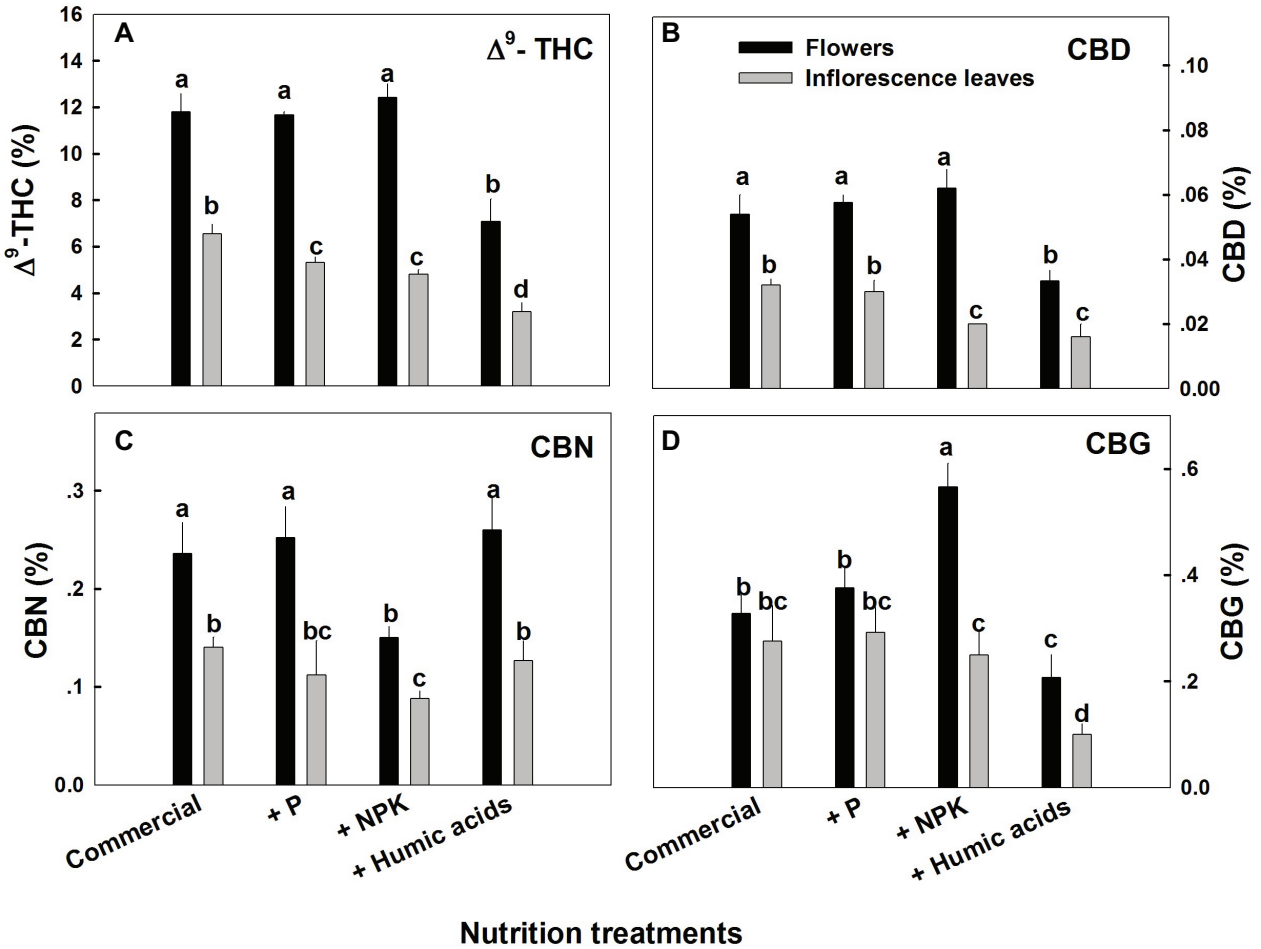
Concentration of major cannabinoids in flowers and inflorescence leaves of medical cannabis plants, as affected by enhanced nutritional supplementation. Δ^9^-THC **(A)**, CBD **(B)**, CBN **(C)**, CBG **(D)**. The top inflorescence of the plant was analyzed. Presented data are averages ± SE (*n* = 6). Different letters above the bars represent significant differences between treatments by Tukey’s HSD test at *α* = 0.05.

**Figure 2:**
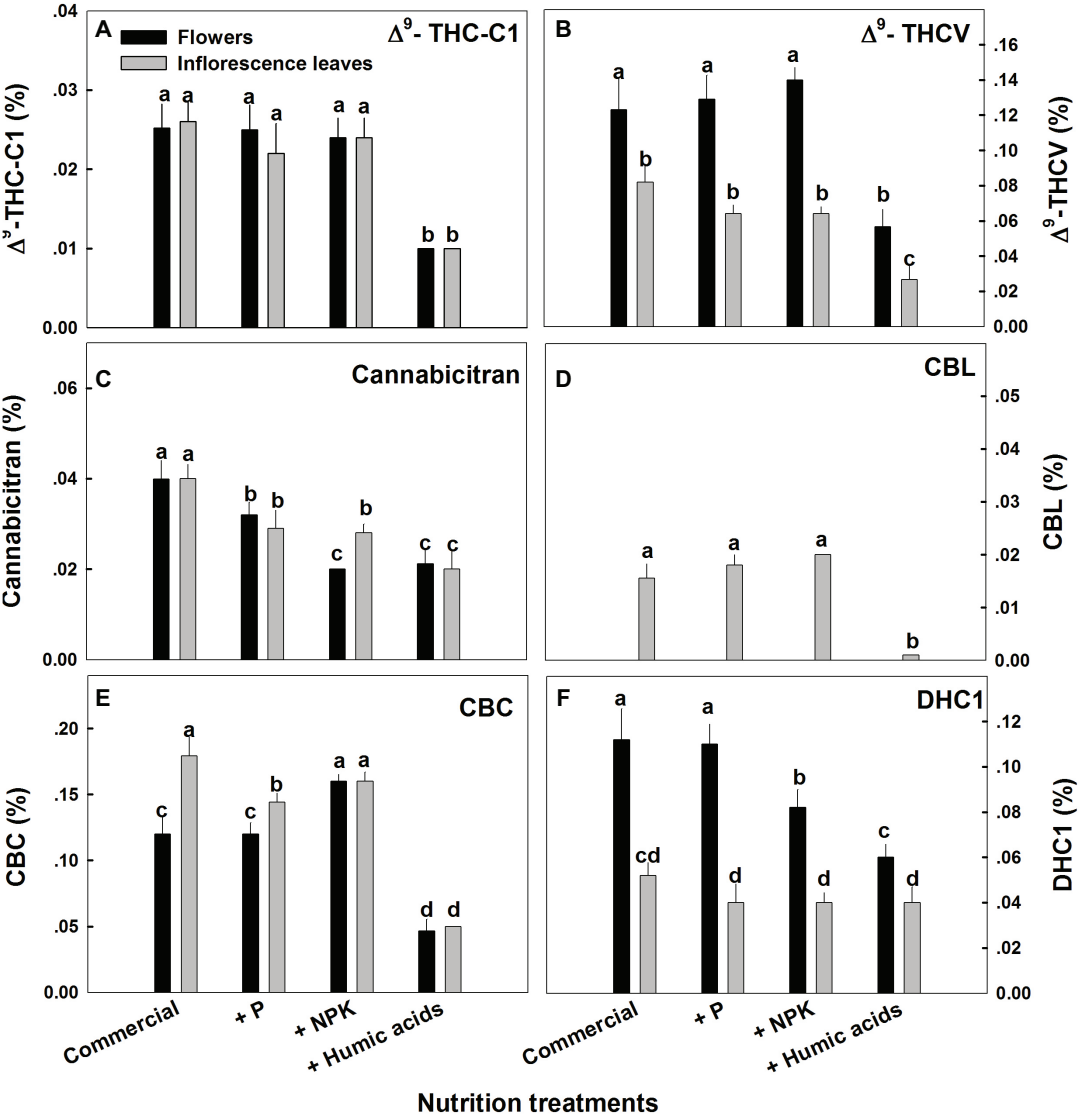
Concentration of minor cannabinoids in flowers and inflorescence leaves of medical cannabis plants, as affected by enhanced nutritional supplementation. Δ^9^-THC-C1 **(A)**, Δ^9^-THCV **(B)**, CBT **(C)**, CBL **(D)**, CBC **(E)**, DHC1 **(F)**. The top inflorescence of the plant was analyzed. Presented data are averages ± SE (*n* = 6). Different letters above the bars represent significant differences between treatments by Tukey’s HSD test at *α* = 0.05.

The various nutritional supplements tested (P, NPK, and HA) elicited distinct changes in cannabinoid content in the flowers as well as the inflorescence leaves (Figures 1, 2). These effects were organ and compound specific. For example, while neither P nor NPK treatment altered THC or CBD levels in the flowers, they did in fact lower THC and CBD content in the inflorescence leaves. For example, THC in the inflorescence leaves was reduced by 16 and 19% by P and NPK supplementation, respectively (Figure 1A). The reverse effect was observed for CBG, where although neither P nor NPK treatments affected inflorescence leaf content, NPK did significantly increase CBG levels in flowers by 71% (Figure 1D). NPK lowered CBN levels in both flowers and inflorescence leaves by 38 and 36%, respectively (Figure 1C). Surprisingly, HA lowered THC, CBD, and CBG levels in both flowers and inflorescence leaves. This trend was also observed with the minor cannabinoids (Figure 2), where HA treatment significantly lowered the levels of THC-C1, THCV, CBC, CBL, CBT, and DHC1 in both flowers and inflorescence leaves.

In fact, with the exception of the increased CBG content in the NPK treatment (by 81% compared to the control; Figure 1D), none of the supplementary treatments increased cannabinoid content. Surprisingly, many of the treatments were found to lower cannabinoid content.

**Figure 3:**
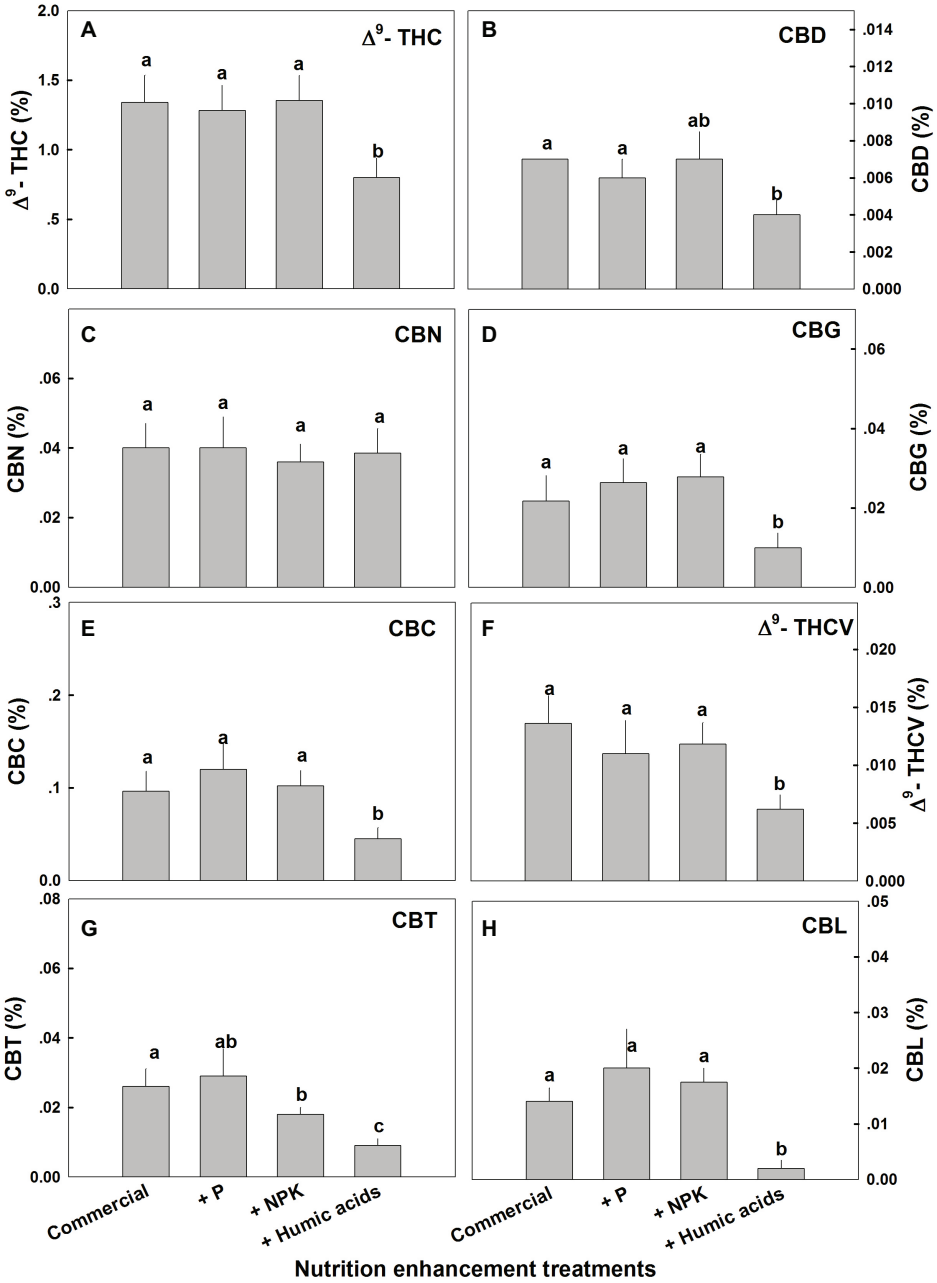
Cannabinoid content in fan leaves of medical cannabis plants, as affected by enhanced nutritional supplementation. Δ^9^-THC **(A)**, CBD **(B)**, CBN **(C)**, CBG **(D)**, CBC **(E)**, Δ^9^-THCV **(F)**, CBT **(G)**, CBL **(H)**. The top inflorescence of the plant was analyzed. Presented data are averages ± SE (*n* = 6). Different letters above the bars represent significant differences between treatments by Tukey’s HSD test at *α* = 0.05.

Similar effects of HA were observed for cannabinoid contents in fan leaves (Figure 3). While P or NPK treatment did not affect the cannabinoid content in fan leaves with the exception of CBCT, which was lowered by NPK treatment by 29%, HA significantly lowered the content of THC, CBD, CBG, CBC, THCV, CBCT, and CBL in fan leaves.

**Figure 4:**
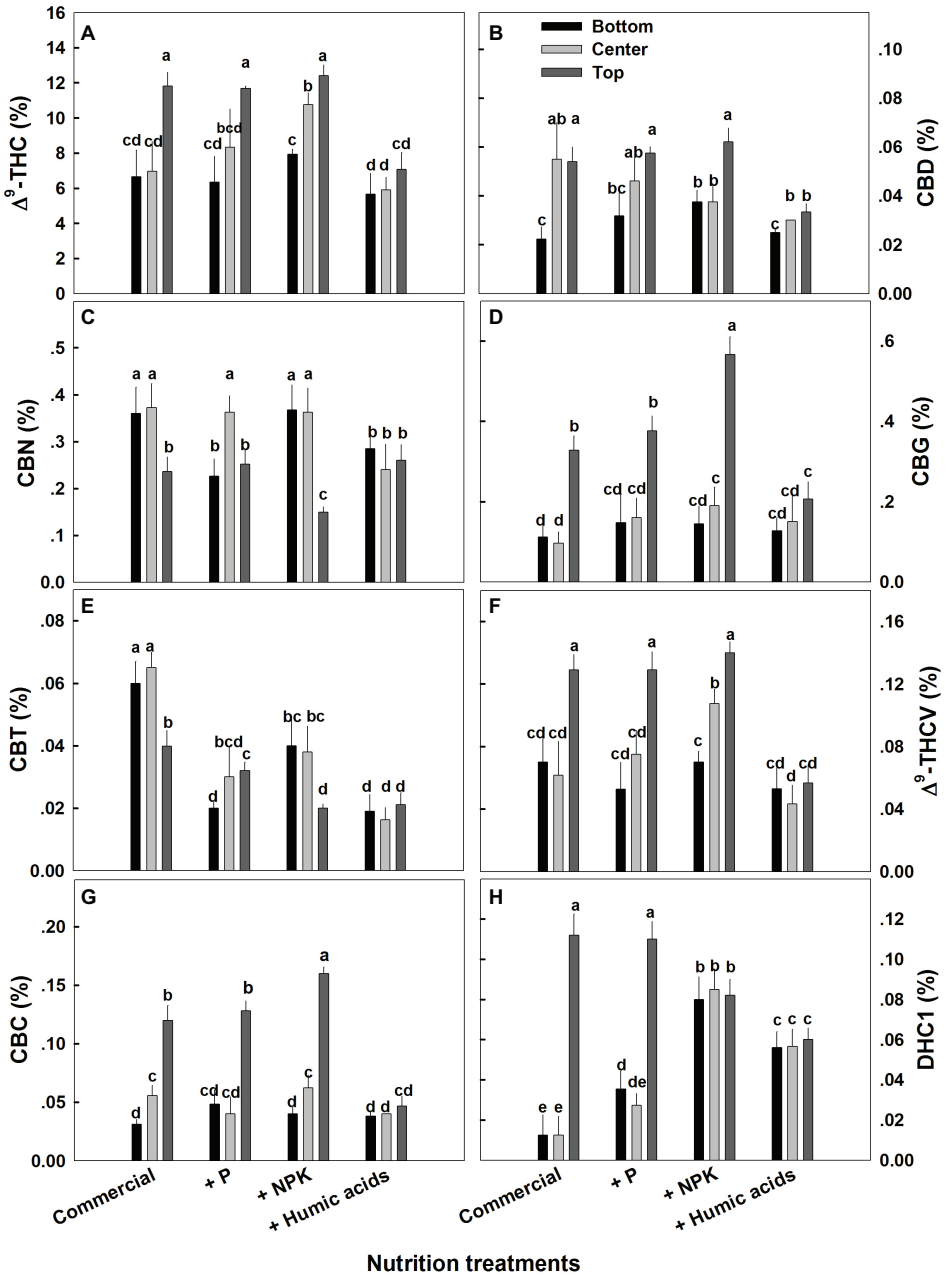
Effect of nutrition supplements on spatial distribution of cannabinoids in the flowers along the cannabis plants. Δ^9^-THC **(A)**, CBD **(B)**, CBN **(C)**, CBG **(D)**, CBC **(E)**, Δ^9^-THCV **(F)**, CBT **(G)**, CBL **(H)**. Inflorescences from the top, center, and bottom of the plant were analyzed. Presented data are averages ± SE (*n* = 6). Different letters above the bars represent significant differences between treatments by Tukey’s HSD test at *α* = 0.05.

The effects of nutritional supplements on cannabinoid content were location dependent (Figure 4). The response to each treatment differed between locations along the plant height. We previously described a natural spatial gradient where THC is more concentrated in the upper regions of the plant (Bernstein et al., 2019). Many other cannabinoids including CBD, CBG, THCV, and CBC displayed a similar trend. In contrast, CBT and CBN were more concentrated in the lower and middle flowers compared to the top ones. In the present study, different nutritional regimes modulate this gradient.

For some of the cannabinoids studied including THC, CBD, and CBG, P supplementation increased the content in the center or bottom of the plant without affecting the levels in the top of the plant (Figure 4). The exception to this trend was seen in CBT, where P actually lowered the CBT content in all parts of the plant. For example, at the bottom of the plant, it was reduced from 0.059 to 0.0195%, and in flowers from the center of the plant, it was reduced from 0.066 to 0.029% (Figure 4E). Similar to P, NPK treatment increased the THC and THCV content in the center of the plant without affecting the top of the plant. In addition, NPK treatment increased the concentrations of CBG and CBC in the top of the plant as well.

Interestingly, HA significantly reduced the natural spatial variability of all of the cannabinoids studied. However, the increased uniformity came at the expense of the higher levels of cannabinoids found in the upper regions of the untreated plants (Figure 4). For example, following HA application THC levels at the top of the plant was reduced from 11.8 to 7.4%, and consequently concentrations throughout the plant height did not differ significantly (Figure 4A).

**Figure 5:**
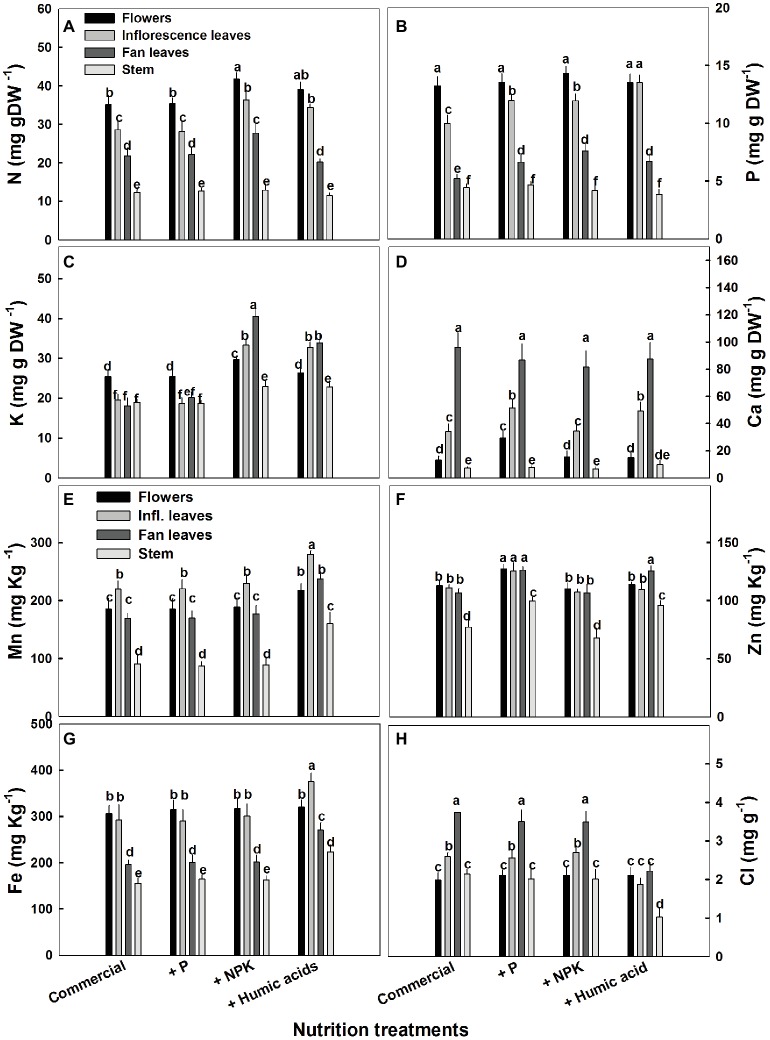
Distribution of macro and micronutrients between plant organs of medical cannabis plants as affected by enhanced nutrition supplements. Concentration of N **(A)**, P **(B)**, K **(C)**, Ca **(D)**, Mn **(E)**, Zn **(F)**, Fe **(G)**, Cl **(H)** in flowers, fan leaves, inflorescence leaves, and stems. Presented data are averages ± SE (*n* = 6). Different letters above the bars represent significant differences between treatments by Tukey’s HSD test at *α* = 0.05.

The influence of the nutritional supplements on mineral levels also varied throughout the plant (Figure 5). Not surprisingly, P treatment increased P levels in the fan and inflorescent leaves. More surprising was the increase in Ca levels in flowers and inflorescence leaves. P supplementation increased Ca levels in the flowers from 13.2 to 29.4 mg g^−1^ (Figure 5D). In addition, P supplementation increased zinc levels in all of the studied organs.

As expected, the NPK treatment increased N, P, and K levels. However, this increase was organ dependent (Figure 5). In inflorescence and fan leaves, a significant increase in N, P, and K was observed, while in flowers, only N and K increased. This is in accord with the lack of effect of P supplementation on P in flowers. In stems, only a small increase in K, from 18.9 to 22.9 mg g^−1^, was observed in the NPK treatment (Figure 5C).

The effects of HA treatment on mineral levels were also organ specific. Surprisingly, in flowers, HA treatment produced no change in mineral content with the exception of Mn, which increased from 185 to 220 mg g^−1^ (Figure 5E). Also unexpectedly, HA did not affect N content in fan leaves with an increase in N levels observed only in inflorescence leaves (from 28 to 34 mg g^−1^, Figure 5A). A significant increase in P levels was observed in HA-treated inflorescence and fan leaves (Figure 5B) and an increase in K was observed in inflorescence and fan leaves as well as in the stem of HA-treated plants (Figure 5C). Both P and HA increased Ca (by 53 and 44%, respectively) in inflorescence leaves (Figure 5D).

**Figure 6:**
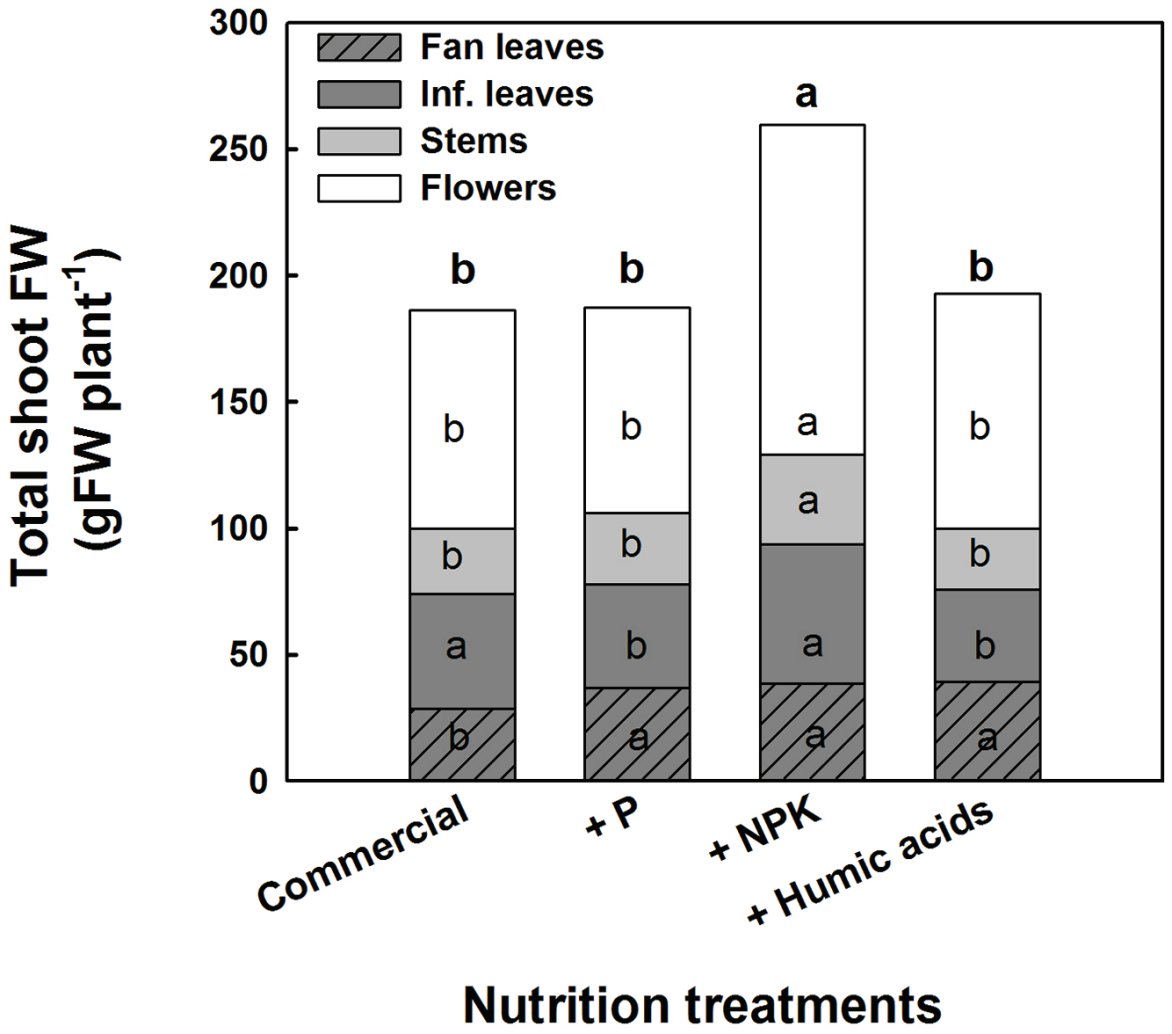
Effect of enhanced nutrition on fresh biomass of shoot organs (fan leaves, inflorescence leaves, stems, and flowers). Data are averages ±SE (*n* = 6). Different small letters above the bars, marked by bold, represent significant difference between treatments in “total shoot biomass.” Across plant part category (i.e., “Fan leaves”, “Inf. leaves”), different small letter inside a bars, represent significant differences within this plant part category, according to Tukey HSD test at *α* = 0.05.

The various nutritional supplements also affected plant growth and the distribution of biomass to the various plant organs (Figure 6). These effects were most notable in the leaves. P, NPK, or HA treatments increased fan leaf biomass. In contrast, P and HA treatments decreased the inflorescence leaves’ biomass by 10 and 13%, respectively. Total shoot biomass was increased by the NPK supplement by 41% as a result of a stimulation of biomass deposition into the flowers and the stems.

The effects of the nutritional supplements on the plant morphological characteristics and growth rates over the course of the flowering period are presented in Table 1. The most pronounced effect was produced by P supplementation, which significantly decreased plant height (by 23.5%) as well as internode and inflorescence length by 0.3 and 1.3 cm, respectively.

**Table 1:** Effects of the nutrition treatments on plant morphological and growth characteristics.

Morphological parameters	Commercial	+ P	+ NPK	+ Humic acids
Plant height (cm)	63.5 ± 2.12 a	48.6 ± 3.2 b	61.1 ± 3.1 a	60.7 ± 2.06 a
Stem diameter (mm)	8.9 ± 0.62 a	9.2 ± 0.4 a	9.6 ± 0.47 a	8.1 ± 0.47 b
Internode length (cm)	1.6 ± 0.06 a	1.3 ± 0.05 b	1.55 ± 0.04 a	1.5 ± 0.09 a
Inflorescence length (cm)	5.4 ± 0.26 a	4.1 ± 0.16 b	5.1 ± 0.35 a	5.5 ± 0.31 a
No. of internodes on the main stem	9.0 ± 0.9 a	9.1 ± 0.97 a	8.6 ± 0.5 a	8.3 ± 0.55 a
No. of side branches on the main stem	7.7 ± 0.92 a	8.8 ± 0.47 a	10 ± 1.15 a	8.8 ± 1.07 a

*Data are averages ±SE (*n* = 6). Different small letters across a row, represent significant differences within the morphological parameter, according to Tukey’s HSD test at *α* = 0.05*.

**Figure 7:**
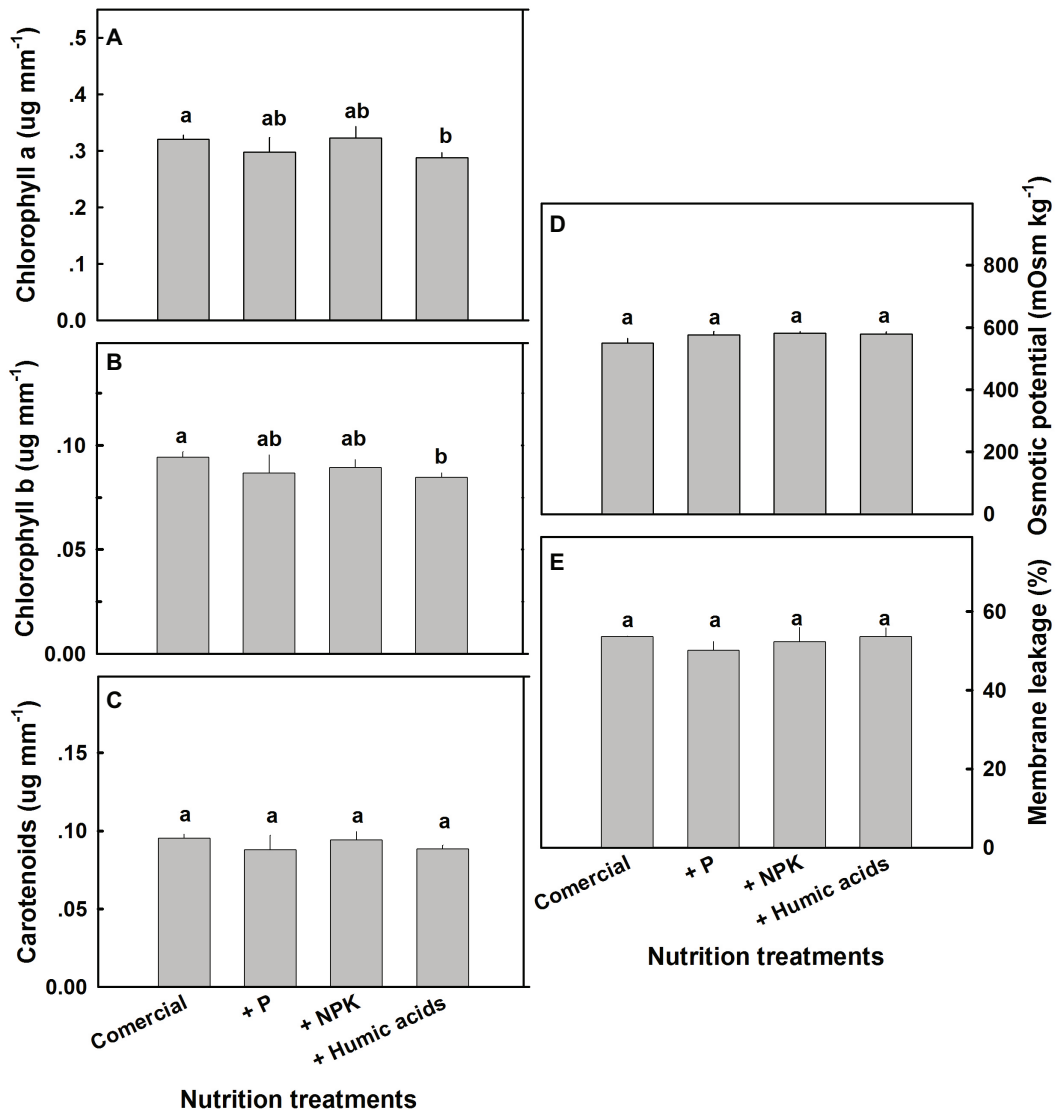
Photosynthetic pigments, osmotic potential, and membrane leakage in cannabis leaves. Chlorophyll a **(A)**, chlorophyll b **(B)**, carotenoids **(C)**, osmotic potential **(D),** and membrane leakage **(E)** of fan leaves. Data are averages ±SE (*n* = 6). Different small letters above the bars represent significant differences according to Tukey’s HSD test, at *α* = 0.05.

A number of physiological parameters were measured including osmotic potential, membrane leakage, and photosynthetic pigment content. Pigmentation was not greatly affected by the nutritional treatments (Figure 7). Only HA lowered chlorophyll a and b levels. Neither osmotic potential nor membrane leakage was significantly affected by any of the nutritional treatments.

## Discussion

One of the most important factors affecting growth, development, and function of plants is mineral nutrition. Macro and micronutrients play a significant role in all aspects of plant metabolism, and their availability in adequate levels is required for optimal physiological performance. Supplementation of nutrients, and especially the macronutrients N, P, and K, is thereby commonly utilized to facilitate optimal plant development and function. In a medicinal plant such as cannabis, optimization of nutrition should take into consideration effects on secondary metabolism as well. The influence of plant nutrition on the production of secondary metabolites is much less known. Some effects of plant nutrition on secondary metabolite biosynthesis have previously been reported (Gershenzon, 1984) and the availability of N, P, and K was found to affect secondary metabolite biosynthesis and accumulation in plants. In cannabis, as well, increased P supply was reported to elevate CBD and THC concentration (Coffman and Gentner, 1977). However, clear rules on the relationship have not yet been established, and the available information suggests that the effects may be species and compound dependent.

The present study aimed to evaluate the sensitivity of the cannabinoid profile to moderate changes in NPK supply, and to HA supplementation, under sufficient supply of the mineral nutrients. The results demonstrate that the response of medical cannabis to enhanced P supplementation is organ and compound dependent. For example, the concentrations of the major cannabinoids THC, CBD, CBN, and CBG in the flowers from the top of the plant were not affected by the P enhancement treatment (Figure 1). THC concentrations were reduced in the inflorescence leaves (Figure 1), while CBN concentrations were reduced only in the flowers from the lower parts of the plants.

Organ, compound, and spatial specificities of the cannabinoid accumulation were also identified in response to the enhanced NPK and HA treatments (Figures 1, 3). While the nutritional supplements lowered the cannabinoid content, this was accompanied by significantly reduced variability throughout the plants of almost all of the cannabinoids studied.

While the results indeed demonstrate that nutrient supplementation can modulate cannabinoid content in an organ- and location-specific manner, the relationship between cannabinoid content and nutritional supplementation is not very clear. The most obvious connections between mineral nutrition and secondary metabolism have been suggested, including the link between N and the production of bioactive N-containing alkaloids (Höft et al., 1996). However, contradicting results have been reported for the effects of N nutrition on secondary metabolites. While some studies identified an effect of N supplementation on secondary metabolite production (Zheljazkov and Margina, 1996; Rioba et al., 2015), others reported no significant effects (Arabaci and Bayram, 2004; Barreyro et al., 2005). In addition, K and Ca supplementations have been shown to increase phenolic and flavonoid content (Ahmad et al., 2016). P availability has been linked to increased polyphenol content (Nell et al., 2009) but P limitation is also linked to an increase in a number of secondary metabolites including phenylpropanoids, flavonoids, and glucosinolates (Pant et al., 2015). That being said, the little that has been revealed is mainly regarding compounds produced *via* the well-known shikimic or mevalonate biosynthetic pathways. Cannabinoids, being terpenophenolics, are produced *via* an alternative biosynthetic pathway which combines the polyketide and DOXP/MEP pathways (Gagne et al., 2012). The factors which influence these converging pathways have yet to be clearly elucidated and it is not surprising that the link to nutritional status has yet to be determined.

While the process by which they influence cannabinoid content is unclear, the nutritional supplementation treatments clearly affected the concentrations of micro and macronutrients in the plant (Figure 5). Synergistic and antagonistic interactions between nutrient cations or anions in membrane transport through the root cells are well documented. Supplementation of minerals can affect external concentrations and hence uptake rates and the subsequent physiological response of the plant. We identified specific effects of the nutritional supplements on mineral accumulation in the different plant organs in addition to modulation of cannabinoid content. While there were some subtle associations linking changes in cannabinoid content and mineral levels, it is difficult to make clear conclusions on their relationship.

Numerous studies investigated the effect of HA on mineral uptake in plants. Supplementation with HA increases N, P, and K in a range of plant systems including wheat (Safwat et al., 2014), corn (Khaled and Fawy, 2011), and pepper (Akladious and Mohamed, 2018). In the present study, HA supplementation increased concentrations of the macronutrients N, P, K, and Ca, and the micronutrients Mn, Zn, and Fe, in at least one vegetative organ of medical cannabis (leaves or stems) (Figure 5). Effects on flower concentration were found only for Mn. It is possible that the increased accumulation of these metals may elicit the production of cannabinoids. It has been previously reported that treatment with metals including Fe and Cu can increase secondary metabolite production in a number of plants (Gorelick and Bernstein, 2014). However, it has yet to be clearly proved in the case of cannabinoids in cannabis and further work is needed.

The need for additional studies is even more glaring considering what is currently known regarding cannabis nutrition in general. Only a small number of scientific studies have been performed dealing with cannabis nutrition and most of these studies focused on hemp varieties grown for fiber. Regarding hemp, N supplementation produced increased height and biomass (Papastylianou et al., 2018). Interestingly, very little response was observed using P or K fertilization treatments (Aubin et al., 2015). But this information is only mildly relevant to medical cannabis, where the concentration of therapeutic cannabinoids is much more important than total biomass or fiber length.

The nutrition supplements did not affect the developmental stage of the plant, i.e., trichome maturation occurred simultaneously for all treatments, and the effect on the mature plant size was small (Table 1). This supports that the nutritional treatments were mild, and within or near the optimal range for plant growth, as was intended for this study. The body of the plants in the experiment developed mainly under the long-day photoperiod, prior to the initiation of the differential treatments, contributing to the small effects of the treatments on plant biomass. The identified impact on the cannabinoid profile, under these conditions that had but small effects on plant development, points at the potential of small variation in the nutritional status for regulation of secondary metabolism in cannabis.

While the role of mineral nutrition in cannabis plant production has been only partially characterized (Caplan et al., 2017), the effects of nutritional supplementation are much less understood. This is certainly the case with the content and distribution of the various cannabinoids, which have not sufficiently been linked with plant nutrition. We observed how nutritional supplements including HA can reduce the spatial variation usually found in the distribution of cannabinoids throughout the cannabis plant. While it is not clear through what mechanism this effect is produced, it is possible that accelerated degradation of cannabinoids in areas of the plant where they are highly concentrated may be a factor. This seems quite plausible in the case of HA on THC distribution (Figure 4). A reduction in the spatial gradient of THC was associated with a complimentary trend of an increase in the degradation products of THC: CBN and DHC.

As a biostimulant, HA is known to elicit the production of various secondary metabolites. It increased the synthesis of flavonoids and phenolics in chicory (Gholami et al., 2018) and pomegranate (Anari Anaraki et al., 2016). However, we did not observe this effect in the case of cannabis. This may be because cannabinoids are produced *via* a non-mevalonate pathway as previously mentioned and the effects of HA on this pathway have yet to be described.

While the present study investigated a low-CBD variety (<0.1%), significant changes in CBD concentrations were apparent between plant organs (Figure 1), locations along the plant height (Figure 4), and between treatments (Figures 1, 3). It would be interesting to investigate treatment effects in cannabis varieties of different chemotypes, such as high-CBD/low-THC, or high-THC/high-CBD types. While our results suggest that nutritional supplements may aid in standardizing cannabinoid content in cannabis, further work is needed to identify the optimal method for each strain and desired cannabinoid profile, as well as to characterize the plants’ response to a wider and more detailed range of individual nutrient application.

## Conclusions

In the present study, the effects of N, P, K, and humic acid supplementation on medical cannabis were studied. While the relationship between cannabinoid content and nutritional supplementation is not clear, the connection is probably a complex relationship involving a number of related parameters including nutrient availability, plant biosynthetic conditions, and other environmental and physiological signals.

Overall, the nutritional supplements significantly reduced cannabinoid variability throughout the plant, demonstrating the importance of developing agro-techniques for standardization of the chemical profile in the cannabis inflorescences. Most importantly, these results demonstrate the potential of environmental factors including mineral nutrition for regulating the concentrations of specific secondary metabolites in defined locals in the cannabis plant. In the case of medical cannabis, which contains hundreds of secondary metabolites with therapeutic activity for various medical indications, the potential for biosynthetic regulation of a compound in a specific location opens up a new avenue of exploration in the search for chemical standardization.

## Author Contributions

JG and NB designed the study and wrote the manuscript. SK conducted the physiological, chemical, and data analyses. RZ controlled the cultivation scheme.

### Conflict of Interest Statement

The authors declare that the research was conducted in the absence of any commercial or financial relationships that could be construed as a potential conflict of interest.

## References

[ref1] Adam D. (2001). Nutritionists question study of organic food. Nature 412:666. 10.1038/35089192, PMID: 11507595

[ref2] Ahmad P. Abdel Latef A. A. Abd_Allah E. F. Hashem A. Sarwat M. Anjum N. A.. (2016). Calcium and potassium supplementation enhanced growth, osmolyte secondary metabolite production, and enzymatic antioxidant machinery in cadmium-exposed chickpea (*Cicer arietinum* L.). Front. Plant Sci. 7:513. 10.3389/fpls.2016.00513, PMID: 27200003 PMC4847423

[ref3] Akladious S. A. Mohamed H. I. (2018). Ameliorative effects of calcium nitrate and humic acid on the growth, yield component and biochemical attribute of pepper (*Capsicum annuum*) plants grown under salt stress. Sci. Hortic. 236, 244–250. 10.1016/j.scienta.2018.03.047

[ref4] Alexander S. P. H. (2016). Therapeutic potential of cannabis-related drugs. Prog. NeuroPsychopharmacol. Biol. Psychiatry 64, 157–166. 10.1016/j.pnpbp.2015.07.001, PMID: 26216862

[ref5] Anari Anaraki B. Ghasem-Nejad M. Meyghani H. (2016). The effect of soil and foliar nutrition of humic acid on quantitative and qualitative characteristics of *Punica granatum* var. ‘Malas Saveh.’ Agric. Sci. Sustain. Prod. 26, 143–153.

[ref6] Arabaci O. Bayram E. (2004). The effect of nitrogen fertilization and different plant densities on some agronomic and technologic characteristic of Ocimum basilicum L. (Basil). J. Agron. 3, 255–262. 10.3923/ja.2004.255.262

[ref7] Aubin M.-P. Seguin P. Vanasse A. Tremblay G. F. Mustafa A. F. Charron J.-B. (2015). Industrial Hemp response to nitrogen, phosphorus, and potassium fertilization. Cftm 1. 10.2134/cftm2015.0159

[ref8] Barreyro R. Ringuelet J. Agrícola S. (2005). Nitrogen fertilization and yield in oregan (Origanum x applii). Cienc. Invest. Agrar. 32, 34–38. 10.7764/rcia.v32i1.305

[ref9] Bernstein N. Gorelick J. Koch S. (2019). Interplay between chemistry and morphology in medical cannabis. Ind. Crops Prod. 129, 185–194. https://www.sciencedirect.com/science/journal/09266690/129/supp/C

[ref10] Bernstein N. Kravchik M. Dudai N. (2010). Salinity-induced changes in essential oil, pigments and salts accumulation in sweet basil (*Ocimum basilicum*) in relation to alterations of morphological development. Ann. Appl. Biol. 156, 167–177. 10.1111/j.1744-7348.2009.00376.x

[ref11] Bernstein N. Sela S. Dudai N. Gorbatsevich E. (2017). Salinity stress does not affect root uptake, dissemination and persistence of Salmonella in Sweet-basil (*Ocimum basilicum*). Front. Plant Sci. 8:675. 10.3389/fpls.2017.00675, PMID: 28512466 PMC5411819

[ref12] Billard V. Etienne P. Jannin L. Garnica M. Cruz F. Garcia-Mina J. M. (2014). Two biostimulants derived from algae or humic acid induce similar responses in the mineral content and gene expression of winter oilseed rape (*Brassica napus* L.). J. Plant Growth Regul. 33, 305–316. 10.1007/s00344-013-9372-2

[ref502] Canellas L. P. Olivares F. L. (2014). Chem. Biol. Technol. Agric. 1:3. 10.1186/2196-5641-1-3 PMC10069009 37026154

[ref13] Canellas L. P. Teixeira L. R. L. Dobbss L. B. Silva C. A. Medici L. O. Zandonadi D. B. (2008). Humic acids crossinteractions with root and organic acids. Ann. Appl. Biol. 153, 157–166. 10.1111/j.1744-7348.2008.00249.x

[ref14] Caplan D. Dixon M. Zheng Y. (2017). Optimal rate of organic fertilizer during the flowering stage for cannabis grown in two coir-based substrates. HortScience 52, 1796–1803. 10.21273/HORTSCI12401-17

[ref15] Coffman C. B. Gentner W. A. (1977). Responses of greenhouse-grown *Cannabis sativa* L. to nitrogen, phosphorus, and potassium. Agron. J. 69, 832–836. 10.2134/agronj1977.00021962006900050026x

[ref16] Conselvan G. B. Pizzeghello D. Francioso O. Di Foggia M. Nardi S. Carletti P. (2017). Biostimulant activity of humic substances extracted from leonardites. Plant Soil 420, 119–134. 10.1007/s11104-017-3373-z

[ref501] Dixon R. A. Paiva N. L. (1995). Stress-Induced Phenylpropanoid Metabolism. Plant Cell 7, 1085–1097. https://www.researchgate.net/profile/Valtcho_Jeliazkov_zheljazkov 12242399 10.1105/tpc.7.7.1085 PMC160915

[ref17] du Jardin P. (2015). Plant biostimulants: definition, concept, main categories and regulation. Sci. Hortic. 196, 3–14. 10.1016/j.scienta.2015.09.021

[ref18] ElSohly M. A. Gul W. (2014). “Constituents of *Cannabis sativa*” in Handbook of Cannabis. ed. Pertwee R. (Oxford: Oxford University Press).

[ref19] Gagne S. J. Stout J. M. Liu E. Boubakir Z. Clark S. M. Page J. E. (2012). Identification of olivetolic acid cyclase from *Cannabis sativa* reveals a unique catalytic route to plant polyketides. Proc. Natl. Acad. Sci. USA 109, 12811–12816. 10.1073/pnas.1200330109 22802619 PMC3411943

[ref20] Gershenzon J. (1984). “Changes in the levels of plant secondary metabolites under water and nutrient stress” in Phytochemical Adaptations to Stress. Recent Advances in Phytochemistry (Proceedings of the Phytochemical Society of North America), Vol. 18, eds. Timmermann B. N. Steelink C. Loewus F. A. (Boston, MA: Springer).

[ref21] Gholami H. Saharkhiz M. J. Raouf Fard F. Ghani A. Nadaf F. (2018). Humic acid and vermicompost increased bioactive components, antioxidant activity and herb yield of Chicory (Cichorium intybus L.). Biocatal. Agric. Biotechnol. 14, 286–292. 10.1016/j.bcab.2018.03.021

[ref22] Gorelick J. Bernstein N. (2014). Elicitation: an underutilized tool in the development of medicinal plants as a source of therapeutic secondary metabolites. Adv. Agron. 124, 201–230. 10.1016/B978-0-12-800138-7.00005-X

[ref23] Gorelick J. Bernstein N. (2017). “Chemical and physical elicitation for enhanced cannabinoid production in cannabis” in Cannabis sativa L. - botany and biotechnology. eds. Chandra S. Lata H. ElSohly M. A. (Cham: Springer International Publishing), 439–456.

[ref24] Grotenhermen F. Müller-Vahl K. (2012). The therapeutic potential of cannabis and cannabinoids. Dtsch. Arztebl. Int. 109, 495–501. 10.3238/arztebl.2012.0495, PMID: 23008748 PMC3442177

[ref25] Gümüş İ. Şeker C. (2015). Influence of humic acid applications on soil physicochemical properties. Solid Earth Discuss. 7, 2481–2500. 10.5194/sed-7-2481-2015

[ref26] Hanuš L. O. Meyer S. M. Muñoz E. Taglialatela-Scafati O. Appendino G. (2016). Phytocannabinoids: a unified critical inventory. Nat. Prod. Rep. 33, 1357–1392. 10.1039/c6np00074f, PMID: 27722705

[ref27] Höft M. Verpoorte R. Beck E. (1996). Growth and alkaloid contents in leaves of Tabernaemontana pachysiphon stapf (Apocynaceae) as influenced by light intensity, water and nutrient supply. Oecologia 107, 160–169. 10.1007/BF00327899, PMID: 28307301

[ref28] Ievinsh G. Vikmane M. Iirse A. Karlsons A. (2017). Effect of vermicompost extract and vermicompost-derived humic acids on seed germination and seedling growth of hemp. Proc. Latv. Acad. Sci., Sect. B 71, 286–292. 10.1515/prolas-2017-0048

[ref500] Jeliazkov V. D. Margina A. (1996). Effect of increasing doses of fertilizer application on quantitative and qualitative characters of mint. Acta horticulturae 426, 579–592. 10.17660/ActaHortic.1996.426.63

[ref29] Khaled H. Fawy H. (2011). Effect of different Levels of humic acids on the nutrient content, plant growth, and soil properties under conditions of salinity. Soil Water Res. 6, 21–29. 10.17221/4/2010-swr, PMID: 23380677

[ref30] Lichtenthaler H. Wellburn A. (1983). Determinations of total carotenoids and chlorophylls b of leaf extracts in different solvents. Biochem. Soc. Trans. 11, 591–592. 10.1042/bst0110591

[ref32] Muscolo A. Sidari M. Nardi S. (2013). Humic substance: relationship between structure and activity. Deeper information suggests univocal findings. J. Geochem. Explor. 129, 57–63. 10.1016/j.gexplo.2012.10.012

[ref33] Nell M. Vötsch M. Vierheilig H. Steinkellner S. Zitterl-Eglseer K. Franz C. (2009). Effect of phosphorus uptake on growth and secondary metabolites of garden sage (Salvia officinalis L.). J. Sci. Food Agric. 89, 1090–1096. 10.1002/jsfa.3561

[ref34] Pant B. D. Pant P. Erban A. Huhman D. Kopka J. Scheible W. R. (2015). Identification of primary and secondary metabolites with phosphorus status-dependent abundance in Arabidopsis, and of the transcription factor PHR1 as a major regulator of metabolic changes during phosphorus limitation. Plant Cell Environ. 38, 172–187. 10.1111/pce.12378, PMID: 24894834

[ref35] Papastylianou P. Kakabouki I. Travlos I. (2018). Effect of nitrogen fertilization on growth and yield of industrial hemp (*Cannabis sativa* L.). Not. Bot. Horti Agrobot. Cluj-Napoca 46, 197–201. 10.15835/nbha46110862

[ref36] Peña-Méndez E. M. Havel J. Patočka J. (2005). Humic substances--compounds of still unknown structure: applications in agriculture, industry, environment, and biomedicine. J. Appl. Biomed. 3, 13–24. 10.32725/jab.2005.002

[ref37] Piccaglia R. Marotti M. Galletti G. C. (1989). Effect of mineral fertilizers on the composition of Salvia officinalis oil. J. Essent. Oil Res. 1, 73–83. 10.1080/10412905.1989.9697754

[ref38] Rioba N. B. Itulya F. M. Saidi M. Dudai N. Bernstein N. (2015). Effects of nitrogen, phosphorus and irrigation frequency on essential oil content and composition of sage (*Salvia officinalis* L.). J. Appl. Res. Med. Aromat. Plants 2, 21–29. 10.1016/j.jarmap.2015.01.003

[ref39] Sacks M. Bernstein N. (2011). Utilization of reclaimed wastewater for irrigation of field-grown melons by surface and subsurface drip irrigation. Isr. J. Plant Sci. 59, 159–169. 10.1560/IJPS.59.2-4.159

[ref40] Safwat H. El-Bassiouny M. Bakry B. A. Abd A. Attia E.-M. Mohamed M. (2014). Physiological role of humic acid and nicotinamide on improving plant growth, yield, and mineral nutrient of wheat (Triticum durum) grown under newly reclaimed sandy soil. Agric. Sci. 5, 687–700. 10.4236/as.2014.58072

[ref41] Schiavon M. Pizzeghello D. Muscolo A. Vaccaro S. Francioso O. Nardi S. (2010). High molecular size humic substances enhance phenylpropanoid metabolism in maize (*Zea mays* L.). J. Chem. Ecol. 36, 662–669. 10.1007/s10886-010-9790-6, PMID: 20480387

[ref42] Shoresh M. Spivak M. Bernstein N. (2011). Involvement of calcium-mediated effects on ROS metabolism in the regulation of growth improvement under salinity. Free Radic. Biol. Med. 51, 1221–1234. 10.1016/j.freeradbiomed.2011.03.036, PMID: 21466848

[ref43] Singh P. Misra A. (2000). Influence of graded level of iron on growth and essential oil production in *Mentha spicata* L. J. Med. Aromat. Plant Sci. 22, 557–562.

[ref420] Zandonadi D. B. Canellas L. P. Fananha A. R. (2007). Indolacetic and humic acids induce lateral root development through a concerted plasmalemma and tonoplast H+ pumps activation. Planta 225, 1583–1595. 10.1007/s00425-006-0454-2, PMID: 17180358

[ref44] Zheljazkov V. Margina A. (1996). Effect of increasing doses of fertilizer application on quantitative and qualitative characters of mint. Acta Hortic., 426, 579–592. 10.17660/ActaHortic.1996.426.63

